# Knowledge, attitudes, and practices regarding malaria control among the slash and burn cultivators in Rangamati Hill tracts of Bangladesh

**DOI:** 10.1186/s12936-019-2849-0

**Published:** 2019-06-25

**Authors:** Avijit Saha, Malabika Sarker, Moktadir Kabir, Guangyu Lu, Olaf Müller

**Affiliations:** 1 Heidelberg Institute of Global Health, Medical School, Ruprecht-Karls-University, INF 130.3, 69120 Heidelberg, Germany; 2 James P Grant School of Public Health, BRAC University, 68, Shaheed Tajuddin Ahmed Sharani, icddr,b Building, Level 6, Mohakhali, Dhaka, 1212 Bangladesh; 3 BRAC, “BRAC Centre”, 75 Mohakhali, Dhaka, 1212 Bangladesh; 4 Medical College of Yangzhou University, Yangzhou University, Yangzhou, 225001 China

**Keywords:** KAP study, Malaria, Slash and burn cultivator, Risk factors, Bangladesh

## Abstract

**Background:**

Slash and burn cultivators are a significant risk group for malaria in South-East Asia. As envisaged in the National Strategic Plan for Malaria Elimination, Bangladesh aims to achieve zero indigenous malaria transmission by 2030. For the national plan to move from malaria control to malaria elimination, targeting the population of slash and burn cultivators is of overriding importance.

**Methods:**

The study used an explorative mixed method design to investigate the knowledge, attitudes, and practices (KAP) regarding malaria prevention and treatment in an endemic area of Bangladesh. Adult slash and burn cultivators in two sub-districts of the Rangamati District were selected and interviewed. Four focus group discussions were conducted, and this was followed by a cross-sectional quantitative survey with 200 participants.

**Results:**

The respondents’ general knowledge about malaria transmission and modes of prevention and treatment was good. However, there were some gaps regarding knowledge about specific aspects of malaria transmission and in particular about the increased risk associated with their occupation. Despite a much-reduced incidence of malaria in the study area, the respondents perceived the disease as life-threatening and knew that it needs rapid attention from a health worker. Moreover, the specific services offered by the local community health workers for malaria diagnosis and treatment were highly appreciated. Finally, the use of insecticide-treated mosquito nets (ITN) was considered as important and this intervention was uniformly stated as the main malaria prevention method.

**Conclusions:**

The findings from this study on promising KAP characteristics in the slash and burn cultivator population are reassuring that the goal of malaria elimination by the year 2030 can be achieved in Bangladesh.

## Background

Malaria remains a major cause for global morbidity and mortality [1]. Although the increased spending by the international health community has helped in significantly reducing the malaria incidence and mortality over the last 15 years, nearly half the world population still lives in malaria-endemic areas [2]. While the vast majority of the current malaria cases occur in sub-Saharan Africa (SSA), the South East Asia (SEA) region is second with regard to the global malaria burden [2].

**Fig. 1:**
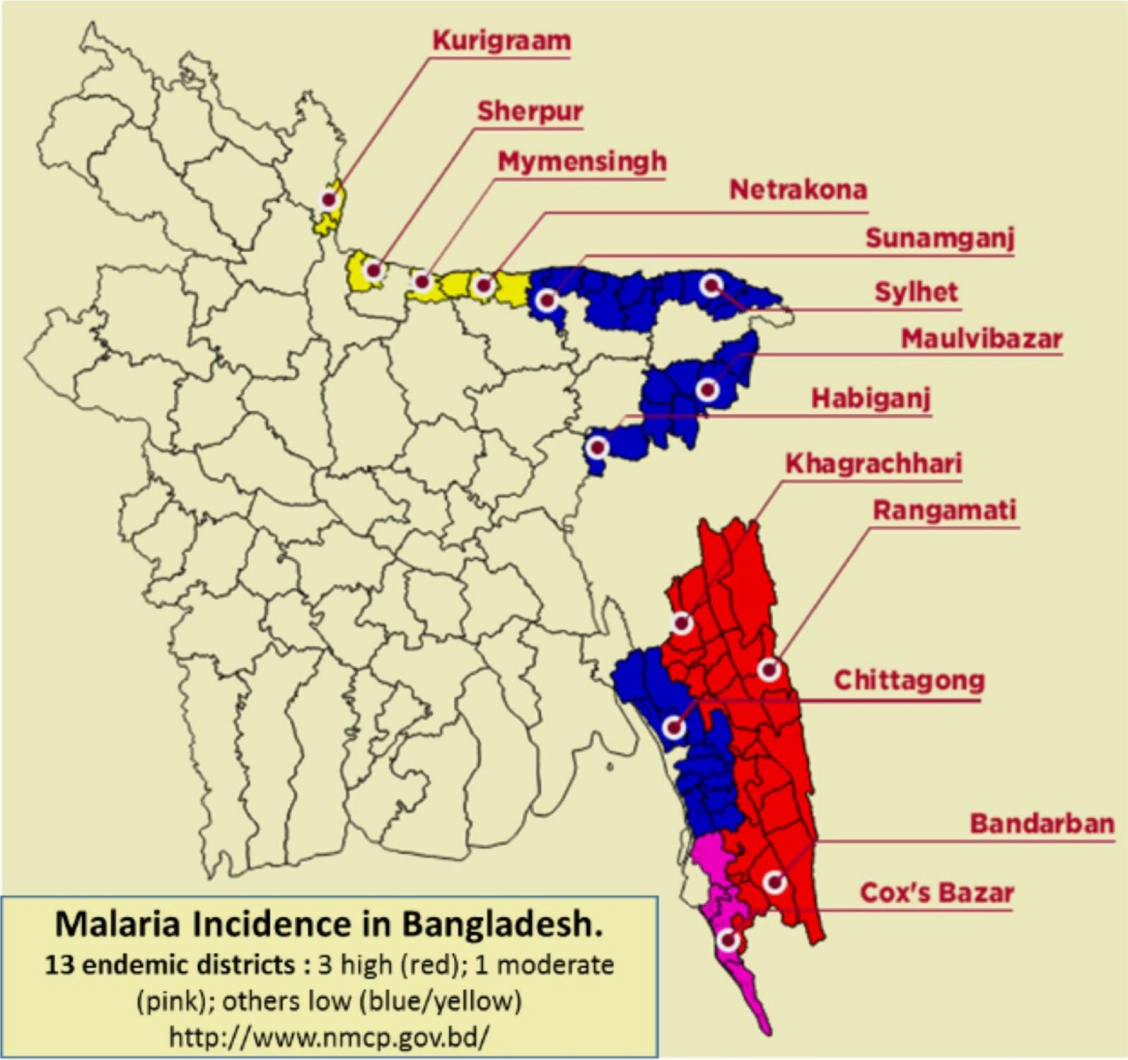
Malaria map of Bangladesh [20]

Bangladesh is situated in southern Asia and bordered by malaria-endemic areas of India and Myanmar. Malaria transmission currently occurs in 13 districts of Bangladesh, which represent 9% of the overall population of 160 million in 2015 (Fig. 1). Three districts are declared as highly endemic, namely Rangamati, Bandarban and Khagrachori in the Chittagong region [3, 4]. A total of 39,719 malaria cases were reported in Bangladesh in 2015 and falciparum malaria accounted for 81% of them [Ministry of Health and Family Welfare (MoHFW), pers. comm.]. Since 2008, the malaria burden has been declining except an upsurge in 2014 which was explained by heavy rains [5, 6].

The National Malaria Elimination Programme (NMEP) of the MoHFW of the Government of Bangladesh aims to achieve malaria elimination (‘zero indigenous transmission’) by 2030, through ensuring equitable and universal access to effective preventive and curative services to all ‘at-risk populations’. The strategy is in-line with both the Strategy for Malaria Elimination in the South East Asia Region (2017–2030) and the Global Technical Strategy for Malaria 2016–2030 and takes into account lessons learned from successful implementation of malaria control efforts in Bangladesh during the past decade [2, 6].

Subsistence agricultural workers, people, working legally or illegally in the forest, and ethnic minority populations living in remote and hard-to-reach areas are the major risk groups for malaria in Bangladesh in these days [7–9]. “Jhum” is a local term used in Bangladesh [10, 11] as well as in parts of India [12] for slash and burn cultivation, which indicates that people do agricultural work in the hillside. In Bangladesh, especially the indigenous people residing in different parts of Chittagong Hill Tracts work as slash and burn cultivators [13]. Slash and burn cultivators regularly work in remote areas and frequently stay overnight which exposes them to the biting of malaria vectors [7]. Therefore, this population is particularly vulnerable and may need special efforts to reduce the malaria infection risk for themselves as well as for the surrounding communities [14].

There is little information about the malaria knowledge, attitudes and practices (KAP) in the population of slash and burn cultivators. KAP studies are a useful method to shed light into aspects relevant to malaria control programmes [15–17]. This study aims to investigate the KAP regarding malaria prevention and treatment by the slash and burn cultivators in Bangladesh.

## Methods

### Study area

The data collection took place in May and June 2017 in Rangamati, one of the hillside districts from the Chittagong Belt in Bangladesh. Rangamati District borders India and is among the districts with the highest malaria incidence in the country (Fig. 1). In the year 2016, a total of 9624 malaria cases were reported from Rangamati, which has a population of about five million [18, 19].

Bangladesh Rural Advancement Committee or at present simply recognized as BRAC, in close collaboration with the national health authorities’ conducts malaria control activities in Rangamati District. Two out of ten sub-districts were selected for data collection for convenience (Rangamati Sadar and Kaptai), because the target group of people was more comfortable to reach and BRAC staffs were available to assist the researcher during the process.

### Study design and procedures

The study adopted an exploratory mixed method design (collection of qualitative and quantitative data) to get an insight into the KAP on malaria prevention and treatment amongst the slash and burn cultivators. In this type of study, usually, the qualitative component precedes the quantitative component and the latter increases the credence degree of the former one [20]. focus group discussions (FGD) were initially conducted to understand the local perception and knowledge about the malaria control programme and also to explore local terms being used for malaria. A quantitative survey was initiated later on in order to build upon the information from the FGDs. Inclusion criteria for participants of both the qualitative and the quantitative study arms were any direct involvement in slash and burn cultivation work in Rangamati District and being over 18 years of age.

Four FGDs were done with each having involved six participants. The participants were selected through a purposive sampling method, to select the information-rich cases [21]. The cross-sectional quantitative survey was conducted after the FGDs were completed and analysed. For the survey, as the target population lives sparsely, and no prior data was available regarding the prevalence or incidence of malaria amongst the target group, a convenience sample of 200 slash and burn cultivators was selected from the study area using the snowball sampling method, a proven method to conduct surveys among hard-to-reach population [22].

The FGDs were conducted by AS with the assistance of a locally recruited translator and a note taker. For the quantitative survey, again two persons were locally recruited and trained by AS to help with the data collection process.

Both, the qualitative interviews and the survey asked questions on knowledge, attitudes, and practice regarding malaria prevention and treatment. The survey used a structured questionnaire with a total of 40 questions. In terms of measuring the financial situation of the study population, the “Progress out of Poverty Index (PPI)” was used to calculate the likelihood of the population being under a national level or international level poverty line [23]. In this study, the 2005 international purchasing power parity poverty line of $2.00/day was considered for measuring the likelihood of people who are either ‘poor’ or ‘non-poor’ [23].

The qualitative data was analysed based on a priori themes in an excel spreadsheet and the quantitative data was analysed using Stata^®^ 13.

### Ethical aspects

Ethical clearance for the study was provided from the Ethical Committee of the Medical School at the University of Heidelberg, Germany, as well as from the Institutional Review Committee of BRAC James P. Grant School of Public Health, BRAC University, Bangladesh. Informed written consent was taken from all participants where all the risks and benefits for participating in the study were mentioned following the Helsinki Declaration [24].

## Results

### Qualitative findings

#### Sociodemographic information

Four FGDs took place, which included a total of 24 adult slash and burn cultivators, 12 females and 12 males, and all belonging to the Chakma Tribal group. The age of participants ranged from 25 to 65 years, and the education level was low (only 7/24 had finished primary school).

#### Jhum cultivation process and local terminology for malaria

Slash and burn cultivators start their work in late spring (March–April) and then continue until the end of autumn (August–September). During this period, they clean the hilly areas to gain fields, they plant the seeds for respective crops, and they sleep near the ground even during rains. Usually, one or two household members work in the field. The eligibility of the household members depends not directly on their age or gender, but on their ability to take part in the whole cultivation process. As they have to stay frequently overnight for protecting the field from animal intrusion, they often build small houses near the area.

*We have to spend around six months in the field starting from April. One month for selection and cleaning the field area and another month for planting seeds in the ground. After that, we have to look after the field continuously. That takes up to six months in total.”* Slash and burn cultivator, FGD 02

“…..*When we go for our Jhum cultivation work, we build ‘macha’ (a local name for small huts) for staying there overnight, if we have to. We build our little houses there, lay down our bed sheets and sleep there”*. Slash and burn cultivator, FGD 02

The respondents denied the use of specific local terms for malaria. However, the kind of responses they gave about malaria disease and its way of transmission was somewhat similar in all of the FGDs. They also mentioned that fever caused by viruses is quite prominent in the area, but they think that it is highly unlikely to be confused with malaria. However, in the past malaria was frequently confused with ‘Kala-jor’ (local dialect for Kala-Azar).

*“No, we call malaria by its original name in here. Sometimes people suffer from fever caused by viruses. But malaria is not known by any other name to our knowledge. In the past, there was a disease named ‘kala*-*jor’ which also caused suffering to people with fever. However, even then we would call malaria by its name only.”* Slash and burn Cultivator, FGD 03

#### Knowledge about malaria

More than two-thirds of the participants knew that a bite from a female mosquito is the way of contracting malaria. Moreover, the common signs and symptoms of the disease were well known by the study subjects.

*“Malaria is caused by the biting of mosquito. I mean, if a female mosquito bites one, then they can contract malaria.”* Slash and burn Cultivator, FGD 04

*“One will have a headache if they have malaria. First, they will have a high fever with chills… After that, for example, they can have seizures. Some people even vomit if the condition gets worse.”* Slash and burn Cultivator, FGD 03

Only a few of the respondents considered their occupation risky for contracting malaria while the majority felt that pregnant women and young children were the main risk groups. Filthy surroundings of households along with puddles or places where polluted and contaminated water can accumulate were some other causes quoted by a majority of FGD participants.

*“Those, who do work like us (Jhum cultivation), roam in the forests to make a living, they have a higher risk of getting malaria. Those who have a dirty household, have bushes around the house, they can also get malaria easily. As mosquitos breed more often in those places, so the risk of malaria also increases in that kind of houses.”* Slash and burn cultivator, FGD 04

#### Attitude towards malaria

Malaria was not considered as frequent any longer and also not as severe for most of the respondents, even for those who had experienced it before. Only two participants mentioned malaria could even cause death if not treated early.

*“We forgot about everything because we haven`t seen new cases for so long. The disease does not occur anymore as it used to in the past. The number of new cases went down so much that we hardly see one or two cases in a year.”* Slash and burn Cultivator, FGD 01

*“… if someone gets malaria and they do not go for treatment or receive treatment early, then they can die. If they take the medicine, this doesn`t become severe. …”* Slash and burn cultivator, FGD 04

Almost half the respondents felt that their workplaces had a low risk of contracting malaria because they clean it for their work and because it is then too windy and open for mosquitoes.

*“… In that place, there are fewer bushes. As the whole place is cleaned by fire, it hardly remains as holt. The place becomes open to air. So, one cannot feel that many mosquitos…. Because mosquitos do not remain in an open*-*air area.”* Slash and burn cultivator, FGD 03

#### Malaria preventive practices

Using mosquito bed nets and cleaning the household surrounding was mentioned as the best malaria prevention method by all the respondents. However, the opinion about who should get priority to sleep under the bed net was rather mixed as some believed everybody should sleep under the nets while others prioritized the elderly, pregnant women and children.

*“Everybody in the family sleeps under the bed net. Nobody is given special preference. If there are pregnant women or children in a family, then they are asked to sleep under the bed*-*nets more than anyone.”* Slash and burn Cultivator, FGD 01

Slash and burn cultivators mentioned taking the ITNs they received for their households along with them whenever they stay overnight for work.

*“Those of us who does Jhum work, we do not get extra bed nets for using at our workplace. So, we take the net that we have in our home with us, then bring it back with us again. Thus we use the bed nets.”* Slash and burn Cultivator, FGD 04

Half of the respondents also mentioned methods like using mosquito repellent coils and creating smoke for mosquito prevention. Also reported was cleaning of household surrounding and trimming of bushes.

*“When extra people are staying at our house, or we do not have enough bed nets for everyone, we create “dhuma” (smoke). We also use mosquito coils.”* Slash and burn Cultivator, FGD 02

The majority of FGD participants mentioned the community health workers as the first line of contact whenever they see signs and symptoms of malaria and for testing their blood. They usually trusted them and were highly satisfied with their services.

“*We seek BRAC’s help for malaria treatment. We have these ‘Didis’ (elder sister) who work in BRAC as health workers. They can give us treatment and medicine….. (therefore) we seek help from ‘didi’ who lives near our home. If they cannot help us, then we go to hospitals with their (community health workers from BRAC) referral, for testing and treatment. They have doctors there, who can do the rest of the treatment.”* Slash and burn Cultivators, FGD 01

*“Their service is excellent. At least it`s better than the service from the hospitals. You can contact them anytime, and you don`t have to spend any money for the services you get. If this is not good then what else.”* Slash and burn Cultivator, FGD 04

### Quantitative findings

#### Socio-demographic characteristics

Two hundred participants took part in the quantitative survey. Table 1 shows the socio-demographic characteristics of the study subjects. 88/200 (44%) respondents were from Rangamati Sadar and 112/200 (56%) from Kaptai. The majority, 118/200 (59%) were males. The mean age of the respondents was 42 years (range 18–70). The majority of the participants had a low educational level, only 45/200 (23%) had finished primary schooling. Roughly half (90/200, 45%) of the participants were the owners of the land which they cultivated, and again roughly half (94/200, 47%) of the participants were defined as poor.

**Table 1:** Socio-demographic characteristics of survey respondents

	Area name		Total N (%)
	Kaptai n (%)	Sadar n (%)	
Gender			
Male	79 (70.54)	39 (44.32)	118 (59.0)
Female	33 (29.46)	49 (55.68)	82 (41.0)
Age (in years)			
18 to ≤ 39	42 (37.5)	47 (53.4)	89 (44.5)
≥ 40	70 (62.5)	41 (46.6)	111 (55.5)
Education level			
No schooling	14 (12.50)	28 (31.82)	42 (21.00)
Unfinished primary	12 (10.71)	18 (20.45)	30 (15.00)
Finished primary	27 (24.11)	18 (20.45)	45 (22.50)
Unfinished secondary	24 (21.43)	12 (13.64)	36 (18.00)
Finished secondary	30 (26.79)	8 (9.09)	38 (19.00)
Higher secondary and above	5 (4.46)	4 (4.55)	9 (4.50)
Own any cultivable land			
No	59 (52.68)	31 (35.23)	90 (45.0)
Yes	53 (47.32)	57 (64.77)	110 (55.0)
Poverty level^a^			
Poor	58 (51.79)	36 (40.91)	94 (47.0)
Non-poor	54 (48.21)	52 (59.09)	106 (53.0)

^a^The Progress out of Poverty Index^®^ (PPI^®^) tool was used in the study to measure the likelihood of the study households to fall below different poverty lines. The tool was validated and weighted using most recent household income and expenditure survey from different countries [20]. The likelihood of people falling under the poverty line was measured using the 2005 international purchasing power parity of less than $2.00/day

#### Knowledge about malaria

Table 2 shows the knowledge of respondents about malaria. The great majority, 178/200 (89%) has heard about malaria and knew that malaria is transmitted through mosquito bites. However, only 91/200 (46%) knew that malaria mosquitoes bite at night. 125/200 (63%) knew the principal signs and symptoms of malaria. Moreover, the majority (164/200, 82%) did know that malaria should be treated with modern medicine and that it should be treated within 24 h of symptom appearance (172/200, 86%).

**Table 2:** Knowledge about malaria

	Area name		Total N (%)
	Kaptai n (%)	Sadar n (%)	
Heard about malaria			
Yes	95 (84.8)	83 (94.3)	178 (89.0)
No	17 (15.2)	5 (5.7)	22 (11.0)
How is malaria transmitted			
By drinking contaminated water	1 (0.9)	1 (1.1)	2 (1.0)
By eating contaminated food	3 (2.7)	0 (0.0)	3 (1.5)
Mosquito bite	94 (83.9)	83 (94.3)	177 (88.5)
Close contact with an infected patient	1 (0.9)	1 (1.1)	2 (1.0)
Don’t know	13 (11.6)	3 (3.4)	16 (8.0)
Knows about basic signs (high fever and chills)			
Yes	76 (67.9)	49 (55.7)	125 (62.5)
No	36 (32.1)	39 (44.3)	75 (37.5)
When do malaria mosquito bites			
During night hours	33 (29.5)	58 (65.9)	91 (45.5)
Any time of the day	55 (49.1)	19 (21.6)	74 (37.0)
Don’t know	24 (21.4)	11 (12.5)	35 (17.5)
What are the treatment options available			
Allopathic treatment (modern medicine)	90 (80.4)	74 (84.1)	164 (82.0)
Self/homeopathic treatment	22 (19.6)	14 (15.9)	36 (18.0)
When to seek treatment			
Within 24 h	87 (77.7)	85 (96.6)	172 (86.0)
Beyond 24 h	25 (22.3)	3 (3.4)	28 (14.0)
Where to seek treatment			
Doctors with a professional degree	48 (42.9)	66 (75.0)	114 (57.0)
No or self-treatment/drug store/traditional healers	64 (57.1)	22 (25.0)	86 (43.0)
Main malaria preventive measures mentioned^a^			
Sleeping under a mosquito net	81 (72.3)	71 (80.7)	125 (76.0)
Clearing the environment from vegetation	87 (77.7)	40 (45.5)	127 (63.5)
Using ITNs	72 (64.3)	46 (52.3)	118 (59.0)

^a^These were multiple choice questions

#### Attitudes toward malaria

Table 3 shows the attitudes of respondents about malaria. They mostly believe that anyone can be infected with malaria and that it is a life-threatening disease. Moreover, the increased risk of sleeping outside and the benefit of sleeping under a mosquito net were well perceived. Finally, there was a positive attitude for getting a complete course of modern malaria treatment.

**Table 3:** Attitudes towards malaria

	Responses			
	Strongly disagree n (%)	Disagree n (%)	Agree n (%)	Strongly agree n (%)
Anyone can be infected with malaria	2 (1.0)	32 (16.0)	121 (60.5)	45 (22.5)
Only children and expectant women have the risk of contracting malaria	5 (2.5)	160 (80.0)	34 (17.0)	1 (0.5)
Thinks malaria as a life-threatening disease	3 (1.5)	22 (11.0)	133 (66.5)	42 (21.0)
During night time, sleeping under mosquito net can prevent malaria	3 (1.5)	29 (14.5)	138 (69.0)	30 (15.0)
Working and sleeping overnight in the garden or forest can increase the threat of getting malaria	3 (1.5)	31 (15.5)	154 (77.0)	12 (6.0)
It is dangerous not to take malaria medicine completely	2 (1.0)	22 (11.0)	119 (59.5)	57 (28.5)
I feel like I should visit the health centers to get my blood tested if I suspect malaria	1 (0.5)	22 (11.0)	147 (73.5)	30 (15.0)
Recovering from malaria without getting any treatment is possible	38 (19.0)	134 (67.0)	23 (11.5)	5 (2.5)

#### Malaria prevention and treatment practice

Table 4 shows the practice of respondents about malaria prevention and treatment. Sleeping under a mosquito net was mentioned to happen very frequently, whereas repellents and mosquito sprays were rather rarely used. Moreover, cleaning the near environment from vegetation and stagnant water was practiced regularly. With regard to treatment in case of an illness, the community health workers were frequently attended while the health center was not regularly visited.

**Table 4:** Malaria prevention and treatment practice

	Responses		
	Always n (%)	Sometimes n (%)	Never n (%)
How often do you sleep under a mosquito net?	145 (72.5)	52 (26.0)	3 (1.5)
How often do other members of the household sleep under mosquito nets?	139 (69.5)	54 (27.0)	7 (3.5)
How often do you check for holes/repair mosquito nets	39 (19.5)	134 (67.0)	27 (13.5)
How often do you use mosquito repellent coils on your house?	15 (7.5)	59 (29.5)	126 (63.0)
How often do you use the anti-mosquito spray in your house?	4 (2.0)	35 (17.5)	161 (80.5)
How often do you clean/cut bushes around your house?	95 (47.5)	86 (43.0)	19 (9.5)
How often do you clean stagnant water near your house?	85 (42.5)	103 (51.5)	12 (6.0)
How often do you visit the health center when you fall sick?	17 (8.5)	168 (84.0)	15 (7.5)
How often do you receive visits from the community health workers?	71 (35.5)	103 (51.5)	26 (13.0)

## Discussion

The main findings from this study provide evidence for a rather good knowledge of malaria in the main risk group for this disease in Bangladesh—the slash and burn cultivator population. This supports the effectiveness of the information/education/communication (IEC) programme provided on malaria by the MoHFW in the endemic areas of Bangladesh [3]. However, the degree of knowledge about the epidemiology of malaria reported in this study is higher compared to what has been reported from other community-based studies in Bangladesh [25, 26]. There were also some misperceptions reported by the slash and burn cultivators concerning specific aspects of the epidemiology of malaria (e.g. biting time of malaria mosquitoes); similar misconceptions were also reported from other countries [17]. A significant problem appears to be the attitude of parts of the slash and burn cultivator population that they are not at a particular risk for malaria infection, which needs to become better addressed through the national malaria programme. However, despite the opinion of the respondents that their work does not expose them too much to mosquito bites and the perception that malaria is no longer the main cause for febrile disease in their working area, they still perceived malaria as a life-threatening disease which needs rapid attention by a health worker. In this regard, it was also reassuring that the presence and the services of the village health workers were highly appreciated in the study population.

Interestingly, no local terminologies for malaria seem to be used in the study area which is quite different from many of the highly endemic areas of SSA and SEA, where this phenomenon is frequently associated with delays in treatment-seeking [27–31].

Protection with bed nets and in particular with ITNs was known to be effective and this intervention was reported to be regularly used within this population of slash and burn cultivators. Reported ITN use was higher than in comparable studies from neighboring countries [32–35]. This might be due to the intense advertisement of this intervention by the government health authorities in Bangladesh as well as by the dedicated work on malaria control of NGOs like BRAC in the study area [36]. This demonstrates that Bangladesh is already on track regarding the goal of the national malaria programme “to ensure equitable and universal accesses to effective preventive and curative services to all at-risk populations”. However, it needs to be considered that out of the four primary malaria vectors in Bangladesh—*Anopheles baimai*, *Anopheles philippinensis, Anopheles sundaicus,* and *Anopheles minimus* sensu lato (s.l.) [37]—the two more exophagic species *An. baimai* and *An. minimus* s.l. were shown to be the main vectors in the study area [38]. The higher outdoor-biting tendency of these vectors indicates that methods which prevent malaria transmission mainly indoors (such as ITNs) might not provide enough protection for the populations living in these remote areas of Bangladesh [38].

Using mosquito repellent coils and creating smoke for mosquito prevention was also mentioned as a tool used to protect from mosquitoes by the participants. However, the evidence for an efficacy of using mosquito repellents is rather limited for populations living in remote malaria endemic areas [39].

The study has some potential limitations. Study participants had a positive attitude towards the national malaria control programme, which may have influenced their practice in malaria prevention and treatment. It concerned both their uptake of preventive interventions as well as their treatment-seeking behaviour and even their reported compliance with full courses of malaria drugs. Moreover, the snowball sampling method used for the recruitment of study participants could have introduced a systematic bias as only other members of the respective cultural group may have been recommended.

## Conclusions

This study has shown a high level of knowledge about malaria as well as a positive attitude towards malaria control interventions accompanied by promising malaria prevention and treatment-seeking behavior among a representative sample of slash and burn cultivators in Bangladesh. It has furthermore shown a good collaboration between governmental and non-governmental actors of the national malaria programme in one of the remaining endemic areas of the country. It is reassuring that the goal of malaria elimination in Bangladesh by the year 2030 appears to be realistic.

## Data Availability

The datasets used and/or analysed during the current study are available on reasonable request. Availability of data can be requested by contacting avijitsahadip@gmail.com or olaf.mueller@urz.uni-heidelberg.de.

## Abbreviations

ITNs: insecticide-treated mosquito nets
KAP: knowledge, attitudes, and practices
SSA: sub-Saharan Africa
SEA: South East Asia
NMEP: National Malaria Elimination Programme
MoHFW: Ministry of Health and Family Welfare
BRAC: Bangladesh Rural Advancement Committee
FGD: focus group discussions
PPI: Progress out of Poverty Index
IEC: information/education/communication
NGO: non-governmental organization
